# Total (fumarolic + diffuse soil) CO_2_ output from Furnas volcano

**DOI:** 10.1186/s40623-015-0345-5

**Published:** 2015-10-26

**Authors:** M. Pedone, F. Viveiros, A. Aiuppa, G. Giudice, F. Grassa, A. L. Gagliano, V. Francofonte, T. Ferreira

**Affiliations:** 1 DiSTeM, Università di Palermo, via Archirafi, 36, Palermo, 90123 Italy; 2 Istituto Nazionale di Geofisica e Vulcanologia, Sezione di Palermo, via Ugo La Malfa, 153, Palermo, 90146 Italy; 3 Centro de Vulcanologia e Avaliação de Riscos Geológicos, University of the Azores, Rua Mãe de Deus, Ponta Delgada, 9501-801 Portugal

**Keywords:** Carbon dioxide flux, Fumaroles, Soil diffuse degassing, Furnas volcano

## Abstract

Furnas volcano, in São Miguel island (Azores), being the surface expression of rising hydrothermal steam, is the site of intense carbon dioxide (CO_2_) release by diffuse degassing and fumaroles. While the diffusive CO_2_ output has long (since the early 1990s) been characterized by soil CO_2_ surveys, no information is presently available on the fumarolic CO_2_ output. Here, we performed (in August 2014) a study in which soil CO_2_ degassing survey was combined for the first time with the measurement of the fumarolic CO_2_ flux. The results were achieved by using a GasFinder 2.0 tunable diode laser. Our measurements were performed in two degassing sites at Furnas volcano (Furnas Lake and Furnas Village), with the aim of quantifying the total (fumarolic + soil diffuse) CO_2_ output. We show that, within the main degassing (fumarolic) areas, the soil CO_2_ flux contribution (9.2 t day^−1^) represents a minor (~15 %) fraction of the total CO_2_ output (59 t day^−1^), which is dominated by the fumaroles (~50 t day^−1^). The same fumaroles contribute to ~0.25 t day^−1^ of H_2_S, based on a fumarole CO_2_/H_2_S ratio of 150 to 353 (measured with a portable Multi-GAS). However, we also find that the soil CO_2_ contribution from a more distal wider degassing structure dominates the total Furnas volcano CO_2_ budget, which we evaluate (summing up the CO_2_ flux contributions for degassing soils, fumarolic emissions and springs) at ~1030 t day^−1^.

**Electronic supplementary material:**

The online version of this article (doi:10.1186/s40623-015-0345-5) contains supplementary material, which is available to authorized users.

## Background

Volcano-hosted hydrothermal systems are the source of sizeable carbon dioxide (CO_2_) emissions, either vented by hydrothermal steam vents (Chiodini et al. 1998) or diffusively released by degassing soils (Chiodini et al. 1999; Rogie et al. 2001; Werner et al. 2008). While considerable work has been spent in the past to estimate the soil CO_2_ flux from hydrothermal areas (e.g., Chiodini et al. 1999, 2001a; Hernández et al. 2001; Werner et al. 2008; Viveiros et al. 2010), far less is known on the CO_2_ output from hydrothermal fumarolic vents (Werner et al. 2000; Fridriksson et al. 2006; Aiuppa et al. 2013; Pedone et al. 2014a, b), which are technically more difficult to study. Consequently, the total (fumarolic + soil degassing) budget remains unconstrained for most hydrothermal systems in our planet (with a few exceptions; Aiuppa et al. 2013).

The aim of the present work is to provide more information on the fumarolic vs. diffusive contribution to the hydrothermal CO_2_ output. Our test site is Furnas volcano, a quiescent polygenetic volcano located in the eastern part of São Miguel island, in the Azores, an archipelago of nine volcanic islands located in the North Atlantic Ocean at the triple junction between American, Eurasian, and Nubian plates (Searle 1980). Furnas volcano has frequently been active in the Holocene (the oldest volcanic products are dated back 100,000 years BP; Moore 1990). The last “magmatic” eruption occurred in 1630 (Cole et al. 1995). In recent times, hydrothermal explosions have re-occurred (in 1840–1841, 1944, and 1990) from the hydrothermal vent (named “Asmodeu”) belonging to the Furnas Village fumarolic field (Ferreira, T: Contribuição para o estudo das emanações gasosas associadas a processos de vulcanismo no arquipélago dos Açores, unpublished Master thesis). Hydrothermal activity is widespread on the island and includes soil diffuse degassing areas (Ferreira et al. 2005; Viveiros et al. 2010), steaming ground, thermal springs, cold CO_2_-rich springs, and low-temperature fumaroles (95–100 °C), mostly concentrated inside the Furnas caldera (where three main fumarolic fields are observed; Viveiros et al. 2010; Caliro et al. 2015). Further studies have been done since the early nineties to study CO_2_ diffuse emissions. The first soil CO_2_ surveys (Baubron et al. 1994; Baxter et al. 1999) in the Furnas caldera identified a CO_2_ degassing area in the proximity of Furnas village. Recently, Viveiros et al. (2010, 2012) estimated the soil CO_2_ fluxes emitted from the Furnas volcanic system using the accumulation chamber method (Chiodini et al. 1998); this led to identifying the presence of several diffuse degassing structures (DDS). The diffuse hydrothermal-volcanic CO_2_ output from Furnas volcano (Furnas caldera and the southern Ribeira Quente village area) was estimated at ~968 t day^−1^ (Viveiros et al. 2010), and the groundwater CO_2_ transport was evaluated at ~12 t day^−1^ (Cruz et al. 1999). As for the majority of the hydrothermal system worldwide, the fumarolic CO_2_ output is unknown.

In this study, we use a tunable diode laser spectrometer (TDLS) to estimate, for the first time, the fumarolic output of volcanic/hydrothermal CO_2_ at Furnas volcano. The TDLS technique is based on measuring the absorption of IR radiation (at specific wavelengths) by a target gas, and can suitably be adapted to measure the flux of volcanic CO_2_ from low-temperature hydrothermal manifestations (Pedone et al. 2014a, b), where the use of traditional UV spectroscopy remote-sensing techniques is prevented by the absence of SO_2_. TDLS employs a light source of very narrow line-width that is tunable over a narrow wavelength range. In other words, tunable diode laser (TDL) steams on absorption spectroscopy using a single isolated absorption line of the target species, allowing positive identification and unambiguous measurement of complex gas mixtures. A major disadvantage is that TDLS applications are better suited to accurate measurement of a specific target gas (known to be present in the atmosphere) than for identification of previously unidentified species (Pedone et al. 2014a). In addition, the quality of the measurements can be limited in highly condensed, optically thick fumarolic plumes.

The fumarole observations were complemented by simultaneous soil CO_2_ measurements with an accumulation chamber. This is the first time in which two methodologies are applied together to evaluate their relative CO_2_ contributions to the total CO_2_ output. Our results, while limited to only one single hydrothermal system, offer new information to understand the modes of hydrothermal carbon release. In addition, the sulfur output from the fumarolic system is quantified based on measurement of the fumarole CO_2_/H_2_S ratios (obtained with a Multi-GAS detector).

### Site description

**Fig. 1:**
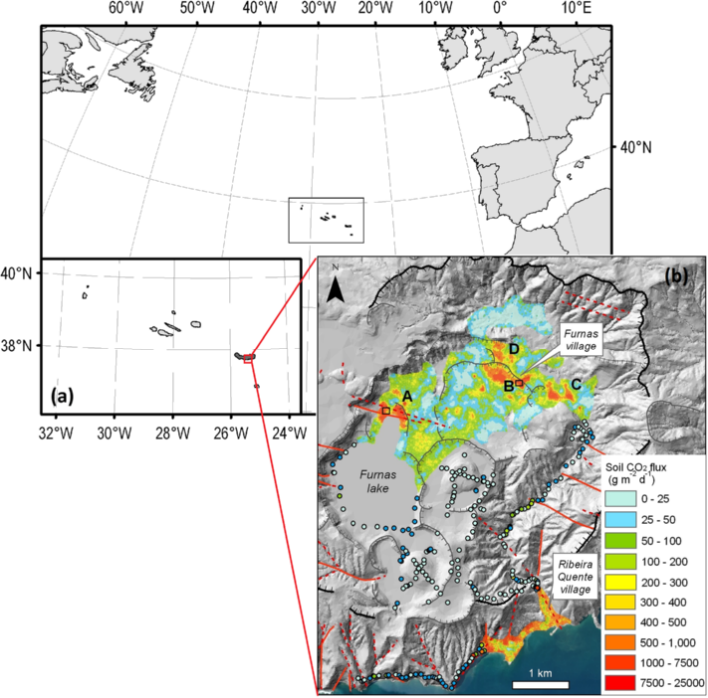
Location of the studied sites. **a** Azores archipelago location and **b** Soil CO_2_ flux map of Furnas volcano (modified from Viveiros et al. 2010). Letters *A*, *B*, *C*, and *D* represent the main diffuse degassing structures defined at Furnas volcano. Letters *A* and *B* (with the associated *black squares*) represent, respectively, Furnas Lake and Furnas Village fumarolic fields, the areas studied in this work. Graded *colored circles* represent soil CO_2_ flux measurement sites that were not used to interpolate the degassing areas (*colored grid*); *red full* and *dashed lines* represent, respectively, observed and inferred tectonic structures; the *bold black lines* represent the older caldera limit and *gray curves* represent crater limits

The two most important fumarolic fields on Furnas are referred to as “Furnas Lake” and “Furnas Village” (see Fig. 1). A small steaming ground exists in the eastern part of the caldera, and the steam emissions are visible in the southern flank of the volcano (Ribeira Quente village).

**Fig. 2:**
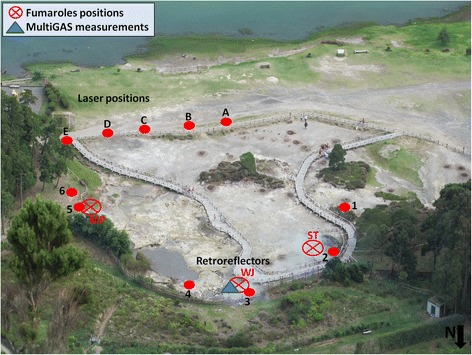
View of Furnas Lake fumarolic site from the top of northern part of the Furnas caldera. The positions of GasFinder unit and retro-reflectors are shown with letters and numbers, respectively. The position of Multi-GAS measurement-point (*blue triangle*) and the degassing vents “ST,” “WJ,” and “MP” (*red crossed-circles*) are also given

**Fig. 3:**
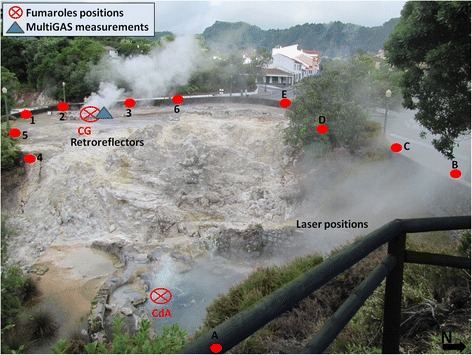
Furnas Village fumarolic site. The positions of GasFinder unit and retro-reflectors are shown with letters and numbers, respectively. The position of Multi-GAS measurement-point (*blue triangle*) and two main degassing vents “CG” and “CdA” (*red crossed-circles*) are also given. *CG*, Caldeira Grande, *CdA* Caldeira do Asmodeu

Our field campaign was carried out in August 2014 in the two main fumarolic fields: Furnas Lake, on the north side of the lake, and Furnas Village, in the eastern side of the caldera (Fig. 1). Figure 2 shows a view of the Furnas Lake area during the campaign carried out on 19 August 2014. The TDL was used to measure the CO_2_ concentration in air close to and/or above three degassing vents (Fig. 2): a vigorously degassing vent with boiling water jet (WJ, close to mirror position 3 in Fig. 2); two smaller fumaroles located in the northwestern part of the area (ST, close to mirror position 2 in Fig. 2); and a mud pool degassing vent (MP, close to mirror position 5 in Fig. 2). Furnas Village (Fig. 3) is the largest fumarolic field in the Furnas caldera. Several moderate to large fumaroles are active within an area of several hundred square meters. “Caldeira Grande” and “Caldeira do Asmodeu” are the main hydrothermal vent manifestations, and are referred to as “CG” and “CdA” in the subsequent text and figures. These fumarolic fields have hydrothermal origin (Ferreira and Oskarsson 1999), with outlet temperatures lower than 100 °C (~97 °C at Lake and ~99 °C at Village; Caliro et al. 2015). The discharge of these fumaroles derive from the shallow hydrothermal aquifers originating from heating of infiltrated local groundwater. The main components are water vapor, carbon dioxide, hydrogen sulfide, nitrogen, hydrogen, oxygen, methane, and argon (Ferreira and Oskarsson 1999; Ferreira et al. 2005; Caliro et al. 2015).

## Methods

Tunable diode lasers are increasingly used in environmental monitoring applications (Gianfrani et al. 1997a) and for volcanic gas observations (Gianfrani et al. 1997b, 2000; De Natale et al. 1998; Richter et al. 2002). Pedone et al. (2014a, b) recently reported on the first direct observations of the volcanic CO_2_ flux by using a portable tunable diode laser (TDL) system.

Like in previous studies (Pedone et al. 2014a, b), we used a GasFinder 2.0 Tunable Diode Laser (produced by Boreal Laser Inc.), a transmitter/receiver unit operating in the 1.3–1.7 μm wavelength range. GasFinder 2.0 is designed to measure CO_2_ concentrations over linear open-paths of <1 km. In order to achieve this, radiation emitted by the IR laser transmitter propagates to a set of gold-plated retroreflector mirrors, where it is reflected back to the receiver and focused onto a photodiode detector. The CO_2_ column amount (in ppm∙m) along each optical path is calculated by the spectral analysis of reflected light, converted into electrical waveform, and processed by using the procedure described in Tulip (1997). CO_2_ column amounts are converted into average CO_2_ concentrations (in ppm) along the path by considering path lengths (measured with an IR manual telemeter, 1 m resolution).

**Fig. 4:**
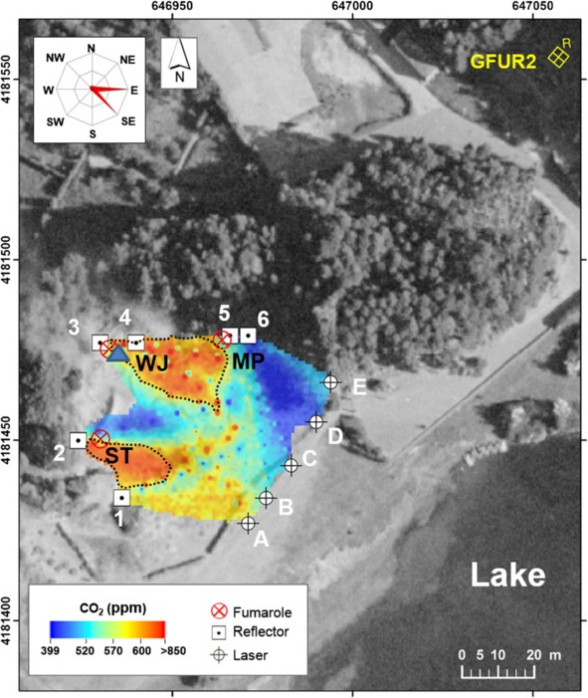
CO_2_ concentrations (ppm) map at “Furnas Lake” survey (using TDL). GasFinder and retro-reflectors positions are shown with letters and numbers, respectively. Investigated fumaroles (WJ, ST, and MP; *red crossed-circles*), Multi-GAS measurement-point (*blue triangle*), and the GFUR2 meteo station positions are given. The principal directions of plume/wind dispersal are given (rose diagram). The CO_2_ flux was estimated integrating the concentration values (>600 ppm) inside the *black dashed lines*, close to the main fumaroles and downwind dispersal direction

**Fig. 5:**
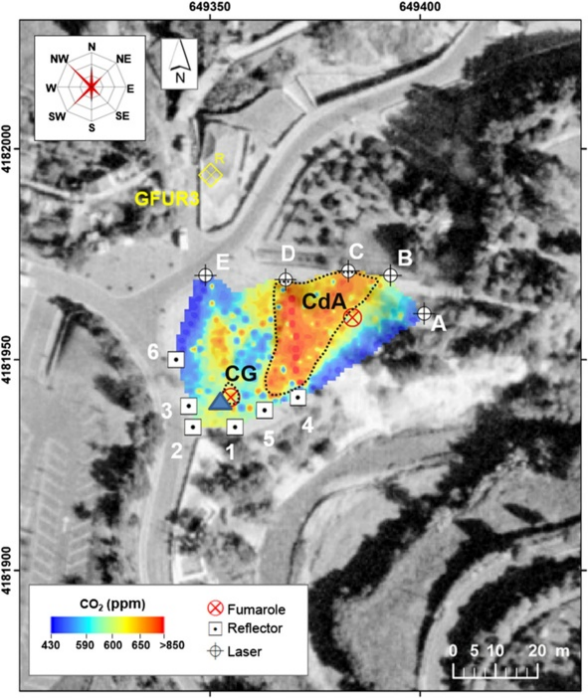
CO_2_ concentrations (ppm) map at “Furnas Village” survey (using TDL). GasFinder and retro-reflectors positions are shown with letters and numbers, respectively. Investigated fumaroles (*red crossed-circles*) and Multi-GAS measurement-point (*blue triangle*), and the GFUR3 meteo station positions are given. The principal directions of plume/wind dispersal are given (rose diagram). The CO_2_ flux was estimated integrating the concentration values (>650 ppm) inside the *black dashed lines*, close to the main fumaroles and downwind dispersal direction. *CG* Caldeira Grande, *CdA* Caldeira do Asmodeu

In the field, the GasFinder was set to measure CO_2_ concentrations at a 1-Hz rate (Pedone et al. 2014a), along an optical (transmitter-retroreflector) path positioned at ~1.20 m height above ground level. Alignment between the laser unit and the retro-reflectors was optimized using a red visible aiming laser and a sighting scope. The GasFinder acquired for several consecutive hours at Furnas Lake (on 19 August 2014) and Furnas Village (on 22 August 2014), totally ~3600 and ~3200 successful readings, respectively. The plumes were fairly transparent during the observations, and the main plume dispersals directions are indicated by rose diagrams in Figs. 4 and 5. Background CO_2_ concentrations were obtained in each of the measurement days/sites by pointing the laser beam toward a mirror, positioned upwind of the fumarolic area. These background values were subtracted in the calculation of integrated column amount (ICA) (see below).

In both days, the position of the GasFinder laser unit was sequentially moved (e.g., from positions A to E in Figs. 2 and 3) to scan the fumaroles’ atmospheric plumes from different angles and viewing directions. The positions of transmitter-receivers were limited by time-logistic constraints (e.g., morphology and accessibility of the degassing areas); given the geometry of the optical paths, we admit some heterogeneities in our CO_2_ maps (Figs. 4 and 5) may result from inhomogeneous, incomplete coverage of the degassing areas. During operations, the GasFinder was left to acquire data along each single GasFinder-retroreflector path for ~3–5 min, before being rotated to measure along the successive path (an additional documentation file shows more details of acquisition-paths (see A1 in Additional file 1).

We considered the possibility of combining the available data to derive a two-dimensional model of CO_2_ distribution, and ultimately—by combination with derived plume vertical transport speeds (Table 1)—to estimate the CO_2_ output from the main investigated fumaroles. The initial step was to use a Matlab script (Pedone et al. 2014a) to obtain a 2D reconstruction of CO_2_ concentrations in the investigated areas, starting from the raw GasFinder dataset. In order to begin calculation, the Matlab script was initialized with the coordinates of GasFinder unit and retroreflector positions (Figs. 2 and 3). The additional input data was a column vector containing the mean CO_2_ column amount (in ppm∙m) obtained for each GasFinder-retroreflector path. With these inputs, the script performed a data inversion using a least-squares method, whose outputs were then processed with a stochastic simulation approach (see Additional file 1: Table S3 and S4), using GSLIB executables (Deutsch and Journel 1998) to obtain the distribution maps of CO_2_ concentrations, shown in Figs. 4 and 5. For each area, the dataset was initially converted by normal score transformation to follow a Gaussian distribution. This normal score-transformed dataset was then used to compute omnidirectional variograms, and finally interpolated with the sequential Gaussian simulations (sGs) method to produce 100 realizations. The maps of Figs. 4 and 5 were produced averaging results of the 100 simulations, using the E-type post-processes method (Cardellini et al. 2003). Grid resolution interpolation parameters are listed in Additional file 1: Table S5. Zonal statistics on the CO_2_ distribution maps was obtained by using the ArcMap 9.3 (ESRI) Spatial Analyst tool (Additional file 1: Table S5).

**Table 1:** CO_2_ fluxes (in t day^−1^) and standard deviation (±1 σ) calculated in the investigated areas based on TDL and accumulation chamber surveys. The plume transport vertical speed (in m s^−1^) is also given for each site. Fumarolic CO_2_ outputs are given for each fumarole emissions (WJ and ST at Furnas Lake; CdA and CG at Furnas Village). Soil CO_2_ flux values are given for the entire areas (Lake and Village, respectively). Total CO_2_ emissions (fumarolic + soil) are also given

Site	Date	Gas speed	CO_2_ flux^a^	CO_2_ flux^b^	Total flux^c^
WJ	19-08	0.98 ± 0.07	17.6 ± 5.3	6.0 ± 0.2	35
ST	19-08	1.00 ± 0.02	11.4 ± 4.7	–	–
CdA	22-08	0.90 ± 0.18	17.8 ± 4.1	3.2 ± 0.2	24
CG	22-08	1.80 ± 0.19	3.00 ± 0.8	–	–

^a^Estimated from GasFinder datasets

^b^Estimated from accumulation chamber datasets

^c^Fumarolic + soil CO_2_ emissions

To convert the CO_2_ maps into a CO_2_ flux, we performed 2D integration inside the areas covered by the fumarolic plumes—the boxes delimited by black dashed lines in Figs. 4 and 5. These integration areas were delimited based on visual (field) observations of plume transport direction, and were mapped so as to include concentration data above fixed concentration thresholds (~600 ppm at lake and ~650 ppm at village). No integration was performed outside these areas, where the CO_2_ contribution was due either to the degassing soils or smaller fumaroles (for which manifestations—as for mud pool “MP”—the gas speed is more difficult to assess). The so-obtained CO_2_ ICAs were multiplied by vertical gas transport speed to obtain a CO_2_ flux (Table 1). Plume vertical transport speed was estimated from recordings of a video camera, pointing toward the fumarolic vents, and acquiring sequences of images of the atmospheric plume at 25 frames per second. The sequences of frames were later post-processed to calculate the time-averaged transport speed of the plume, after converting camera pixels into distances (using a graduated pole, positioned close to the vent).

During the field campaigns, we used a Multi-GAS (Aiuppa et al. 2011, 2012 for information about setup and performance of the instrument) to measure the compositions of the main gas manifestations. In detail, the Multi-GAS was exposed to the atmospheric plumes of the main fumaroles (WJ at Furnas Lake and CG at Furnas Village; blue triangles in Figs. 2, 3, 4, and 5) to measure the concentrations of CO_2_ (by NDIR spectroscopy), SO_2_, and H_2_S (by specific electrochemical sensors). The specific sensors mounted onboard the Multi-GAS were as follows: a Gascard Edinburgh Instruments infrared spectrometer for CO_2_ (0-3000 ppmv range, with a resolution of 0.8 ppmv), a 0- to 200-ppmv SO_2_ electrochemical sensor, and a 0- to 50-ppmv H_2_S electrochemical sensor (all from City Technology, and the manufacturer quoted a resolution of 0.5 ppmv). We also used a temperature-humidity Galltec sensor (*T* range, −30 to 70 °C; Rh range, 0–100 %). The Multi-GAS sensors were calibrated in the laboratory by using standard gas cylinders of concentrations within the sensor ranges (all in nitrogen matrixes). Laboratory tests indicate a typical measurement error in the CO_2_/H_2_S ratios of ≤20 %.

**Fig. 6:**
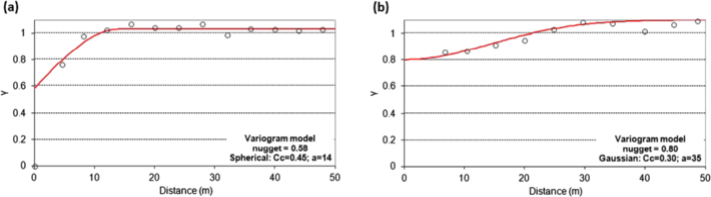
Experimental and modeled variograms of the soil CO_2_ flux normal scores. **a** Furnas Lake. **b** Furnas Village datasets. Legend: Cc (partial sill); a (range, m)

**Fig. 7:**
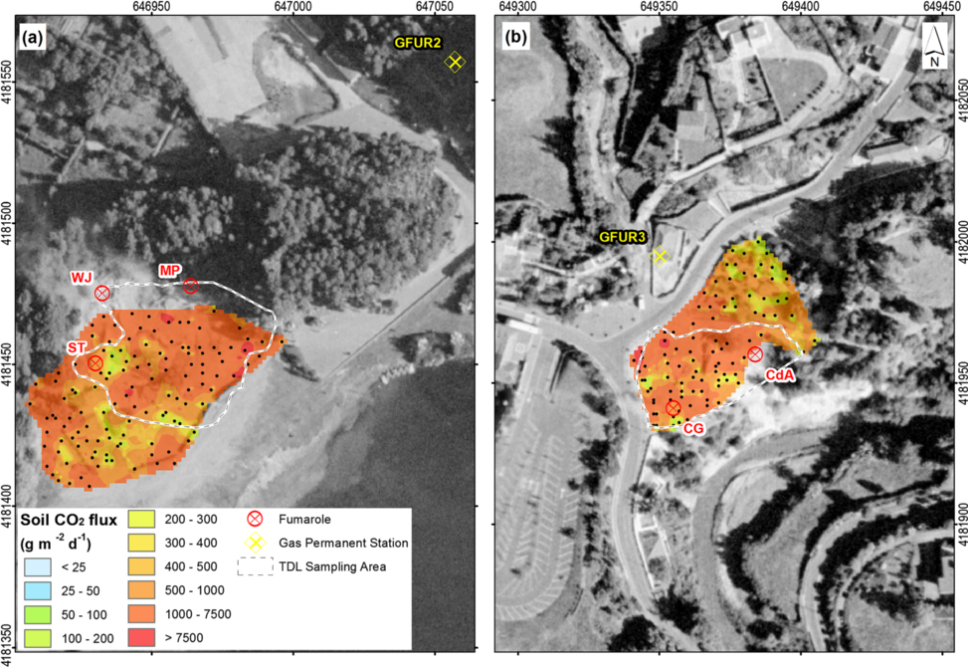
Soil CO_2_ flux distribution maps for Furnas Lake (**a**) and Furnas Village (**b**) fumarolic ground. *Black dots* represent the accumulation chamber sampled points. The fumarolic emissions studied in this work (ST, WJ, and MP at Furnas Lake; CG and CdA at Furnas Village) and the area investigated via TDL (*white dashed lines*) are also shown

In both areas, we also performed a detailed soil CO_2_ flux survey using a portable accumulation chamber (Chiodini et al. 1998). A chamber of a known volume is placed on the soil surface, the variation of CO_2_ concentration is measured, and the CO_2_ flux is calculated from the increase rate of the CO_2_ concentration. The portable chamber is equipped with a LICOR LI-800 infrared CO_2_ detector that measures CO_2_ concentrations in the range 0–2 vol %. Calibration of the instrument was performed in the laboratory prior to field work. Considering that soil gas fluxes may be highly affected by meteorological conditions (e.g., Viveiros et al. 2008), measurements were performed during stable weather conditions and the CO_2_ flux values recorded in a permanent soil CO_2_ flux station (named GFUR2) used as control site; this station is installed close to Furnas Lake fumarolic field (Fig. 4). Soil CO_2_ fluxes in the permanent station showed a coefficient of variation (ratio between standard deviation and average value) of 0.02 and 0.03 during the surveys carried out in 19 and 22 August 2014, respectively, at Furnas Lake and Furnas Village fumarolic fields. At Furnas Lake, a total of 124 measurements were carried out in an area of approximately 3666 m^2^. At Furnas Village, a total of 95 soil CO_2_ flux measurements were carried out in an area of about 2300 m^2^. A sampling space varying between 3 and 5 m was used in these detailed surveys. The grid used was as regular as possible, considering the presence of steam vents, water streams, irregular topography, and some man-made structures in the study sites. The CO_2_ flux values were interpolated using sGs (Deutsch and Journel 1998; Cardellini et al. 2003), which consists in the production of numerous simulations of the spatial distribution of the CO_2_ flux. Considering that data has to follow normal distribution in order to apply sGs, normal score transformation was applied to the original data. Similarly as described above, in this case, omnidirectional variograms of normal scores for the two surveyed areas were also computed (Fig. 6). One-hundred realizations of the flux grid were created for each study site. Parameters used in the sGs procedure are available in A3 section of Additional file 1: Tables S6 and S7. The CO_2_ output was calculated by integrating the average values estimated from sGs over the area. The mean and the standard deviation computed for the 100 realizations are, thus, assumed to be, respectively, the CO_2_ output (Fig. 7) and its uncertainty for each area.

## Results and discussion

### TDL-based CO_2_ distribution maps

Figure 4 is a distribution map of atmospheric CO_2_ concentrations, calculated for a horizontal air cross-section taken at a 1.20-m height above the degassing soil and fumaroles of Furnas Lake (see Fig. 4). The calculated CO_2_ concentration values range from ~390 to >850 ppm. In detail, the lowest CO_2_ concentrations are detected in the eastern portion of the investigated area, far from the fumarolic field (close to laser positions B–E in Fig. 4). In contrast, two main clusters of higher CO_2_ concentrations (>580) are detected on the northern, northwestern, and western portion of the map, in the proximity of the main degassing fumarolic vents (crosses). Peak CO_2_ concentrations (>850 ppm), in particular, are observed close to the fumaroles WJ, MP, and ST (Fig. 4). It is worth noting that the CO_2_ peaks appear in the map as shifted toward the east-southeast relative to the fumaroles’ positions, in agreement with the prevalent plume transport direction during the observations (see wind rose diagram in Fig. 4).

Figure 5 is a similar map derived from interpolation of the GasFinder dataset acquired during operation field at Furnas Village. Again, the CO_2_ concentration anomalies are fairly consistent with the location of the main visible degassing areas. CO_2_ concentrations (~700–800 ppm) peak in a wide degassing area downwind (west of) fumarole CdA (the dominant plume transport directions during the observations were toward NW and SW; Fig. 5). A secondary, more moderate CO_2_ anomaly is observed north of CG fumarole. In contrast, the lowest CO_2_ concentrations are detected along paths A4 and E6, respectively, upwind and/or more remote from the fumaroles (Fig. 5; Additional file 1: Table S2).

### Soil CO_2_ flux degassing

Soil CO_2_ fluxes (derived by using the accumulation chamber method) varied between 6 and 28,231 g m^−2^ day^−1^ in the Furnas Lake area, with a mean value of 1510 g m^−2^ day^−1^. For the Furnas Village fumarolic ground, soil CO_2_ flux average value was 1279 g m^−2^ day^−1^. Minimum and maximum values were, respectively, 5 and 22,154 g m^−2^ day^−1^ in this site (Table 2). Experimental variograms show spherical and Gaussian structures, respectively, for Furnas Lake and Furnas Village datasets (Fig. 6). The high nugget effect associated to Furnas Village (Fig. 6b) detailed survey is probably explained by the soil heterogeneities in this fumarolic ground, where the soil alteration causes large differences in the permeability at small scales. This behavior was previously observed by Viveiros et al. (2010) for data acquired in the same sampling sites. Figure 7 shows the soil CO_2_ flux distribution maps for Furnas Lake and Furnas Village fumarolic ground.

**Table 2:** Comparison between soil CO_2_ fluxes at Furnas Lake and Furnas Village during 19 and 22 August 2014 (this study) and soil CO_2_ fluxes calculated in previous study (March 2008 at Furnas Lake and June–July 2009 at Furnas Village). Areas (in m^2^), soil CO_2_ flux, mean, median, minimum, maximum values (expressed in g m^−2^ day^−1^), and skewness are shown. *Sp* sampling period (year), *Np* number of points

Site	Sp	Np	Area	Mean	Median	Minimum	Maximum	Skewness
Lake	2014	124	3666	1510	266	6	28,231	5
Village	2014	95	2300	1279	248	5	22,154	4
Lake	2008	59	3666	947	187	6	20,579	6
Village	2009	87	2300	448	231	1	3452	3

### Multi-GAS in-plume measurements

The Multi-GAS detected strong volcanic CO_2_ and H_2_S signals in both areas. SO_2_ was undetected (<0.05 ppmv) at both sites, and the relative humidity in the plumes ranged 30–50 % and 40–60 % at Furnas Lake and Village, respectively. The relative humidity variations in the plumes did not systematically correlate with changes in either CO_2_ or H_2_S, precluding the volcanic H_2_O signal to be clearly resolved.

**Fig. 8:**
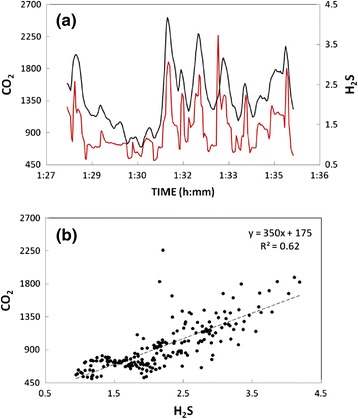
CO_2_ and H_2_S signals detected (by using Multi-GAS) in 215 in-plume measurements close to “WJ” vent at Furnas Lake site. **a** CO_2_ signal (*red curve*) and H_2_S signal (*black curve*) are expressed in ppm. **b** Correlation between two signals. In this plot (~7 min of data acquisition), the CO_2_/H_2_S (molar ratio) is 350 (close to the mean ratio value found in the entire acquired measurements) and the *R* ^2^ regression line is ~0.62

An example of Multi-GAS acquisition is shown in Fig. 8, in which the CO_2_ and H_2_S signals detected in 215 measurements close to “WJ” degassing vent were plotted. At Furnas Lake, the Multi-GAS derived compositions of the plumes of the main jet-water degassing vent (WJ; Figs. 2 and 4) are consistent with literature data based on direct fumarole sampling. We obtain a mean Multi-GAS-based CO_2_/H_2_S plume (molar) ratio of 353, which fits well with the mean ratio of 348 observed in an earlier chemical survey of the fumarole (Ferreira and Oskarsson 1999). At Furnas Village, the mean CO_2_/H_2_S plume ratio of 150 measured by the Multi-GAS at the CG fumarole (Figs. 3 and 5) is not far from the mean fumarolic ratio of 120 of Ferreira and Oskarsson (1999). The CO_2_/S_t_ ratios of Furnas fumaroles (this study) are similar to those seen in other hydrothermal fluids of similar outlet temperatures. For example, the CO_2_/S_t_ ratio detected at Furnas Lake is close to that quoted by Aiuppa et al. (2013) for Pisciarelli fumaroles of Campi Flegrei (CO_2_/S_t_ ratio of 300, range 210–410) and by Chiodini et al. (2001b) for Vesuvius fumaroles (CO_2_/S_t_ ratio of 342). The CO_2_/S_t_ ratio detected at Furnas Village is comparable with compositions of fumaroles at Solfatara crater, Campi Flegrei (CO_2_/S_t_ ratio of 130, range 120–152; Aiuppa et al. 2013) and Ischia (CO_2_/S_t_ ratio of 119; Chiodini et al. 2004).

### CO_2_ and H_2_S output

We estimated the fumarolic CO_2_ output from Furnas volcano by 2D integration of the TDL-based CO_2_ concentration values. This integration was restricted to the shaded box areas of Figs. 4 and 5, downwind the main fumaroles. These integrated CO_2_ amounts within the defined areas were finally multiplied by the vertical gas transport speeds to calculate the fluxes (Table 1). The so-estimated fumarolic CO_2_ fluxes were ~29 t day^−1^ at Furnas Lake (17.6 ± 5.3 t day^−1^ emitted from WJ and 11.4 ± 4.7 t day^−1^ from ST) and ~21 t day^−1^ at Furnas Village (17.8 ± 4.1 t day^−1^ emitted from CdA and 3 ± 0.8 t day^−1^ from CG) (Table 1). The cumulative fumarolic output from the two areas is therefore ~50 t day^−1^.

We additionally infer, using this fumarolic CO_2_ flux (Table 1) and the Multi-GAS-derived CO_2_/H_2_S ratios, fumarolic H_2_S fluxes of 0.08 t day^−1^ (Furnas Lake) to 0.17 t day^−1^ (Furnas Village), respectively. Our results indicate that Furnas fumaroles are weak, but yet not negligible, sources of S. The present-day H_2_S flux of 0.25 t day^−1^ (0.08 + 0.17 t day^−1^) at Furnas is nearly 1 order of magnitude lower than typical emissions from Campi Flegrei volcano (1.5–2.2 t day^−1^; Aiuppa et al. 2013), which similarly display a H_2_S-dominated flux and more than 1 order of magnitude lower than emissions from Vulcano Island (6–9 t day^−1^; Tamburello et al. 2011), whose fumarolic field hosts hot (>400 °C) magmatic fumaroles. For comparison, the H_2_S flux sustained by Etna, the largest source of volcanic gases worldwide, is 200 to 400 times larger (50 to 113 t day^−1^; Aiuppa et al. 2005) (note Etna additionally emits thousands of tons of SO_2_ every day; Caltabiano et al. 2004).

On the basis of the integration of the average of 100 sequential Gaussian simulations (Deutsch and Journel 1998; Cardellini et al. 2003), over the sampled area, mean soil diffuse CO_2_ emissions of ~6 ± 0.2 t day^−1^ and 3.2 ± 0.2 t day^−1^ were estimated for Furnas Lake and Furnas Village degassing areas, respectively. We then obtain a cumulative soil CO_2_ release of ~9.2 t day^−1^. Detailed soil CO_2_ surveys in the Furnas Lake and Furnas Village fumarolic fields (same as Fig. 7 in this study) have previously been performed by Viveiros et al. (2012). For the sake of comparison, we extracted from these earlier surveys (made between 2008 and 2009) the results obtained in the same areas as those investigated in the present study (2014) (Fig. 7). This comparison shows that the Furnas Lake area emitted in 2008 about 3 t day^−1^ of CO_2_, or about half of what was measured in 2014. Similarly, the Furnas Village area was found in 2009 to emit nearly half (1.7 t day^−1^) of the amount of CO_2_ released in 2014 (3.2 ± 0.2 t day^−1^). Increased CO_2_ emissivity in 2014 is consistent with the different average CO_2_ flux values observed in the two different surveys (Table 2) but is not supported by records of the permanent soil CO_2_ flux station GFUR2 (where lower soil CO_2_ flux values—about 250 g m^−2^ day^−1^—were recorded during 2014 compared to 2008 and 2009 surveys—values higher than 350 g m^−2^ day^−1^). We caution that differences in sampling grid/density (that was significantly higher in the present surveys, as can be also observed in the number of points of Table 2) can partially explain the CO_2_ flux diversity between the two campaigns (Viveiros et al. 2010).

### Implications

Our results here suggest that, in the actively degassing fumarolic areas of Furnas, fumarolic vents, with their cumulative fumarolic output of ~50 t day^−1^, dominate the total CO_2_ degassing budget (~59 t day^−1^) and overwhelm the relatively marginal contribution (~15 %) of soil diffuse degassing. While the fumarolic output can be locally important, however, its contribution to the total CO_2_ degassing output becomes marginal at the scale of the entire volcano. Earlier soil CO_2_ flux measurements, in fact, have demonstrated a total diffusive hydrothermal CO_2_ release at Furnas, from an area (6.2 km^2^) far larger than that studied here, as high as 968 t day^−1^ (Viveiros et al. 2010). From this comparison, we conclude that—at Furnas—the most actively degassing areas, although featuring the most visible (e.g., fumaroles, hot pools) and spectacular manifestations of thermal activity, in no way correspond to the areas of largest CO_2_ output: in total, the actively degassing areas contribute only about 5 % to the total CO_2_ output.

To what extent this conclusion can be generalized to other hydrothermal-volcanic systems remains unknown, given the paucity of information we have in hand. A small contribution of fumarolic gas vents to the total CO_2_ budget was also suggested by Fridriksson et al. (2006) for the Reykjanes geothermal area, SW Iceland, where the contribution of steam vents/mud pools is only about 3 % of the soil CO_2_ degassing output. In other systems, in contrast, the contribution of fumaroles is manifestly more significant. At Mud volcano (Yellowstone, USA), for example, Werner et al. (2000) estimated that fumarolic vent emissions contribute to more than 32 % of the total degassing; based on statistical approach, the authors even suggested that the hydrothermal-focused emissions can be responsible for up to 63 % of the total degassing in that thermal area. At Campi Flegrei, the CO_2_ fumarolic output of ~500 t day^−1^ (range, 460–507 t day^−1^, Aiuppa et al. 2013; Pedone et al. 2014a) makes a substantial contribution to the total CO_2_ output, which is still dominated by soil diffuse degassing (~1100 ± 120 t day^−1^; Chiodini et al. 2010). From these examples, we argue that much remains to be done to fully understand the fumarolic vs. diffusive gas contribution at volcano-hydrothermal systems. The relative significance of the fumarolic output can differ significantly from one volcano to another. A general conclusion is that the relative significance of the above two forms of gas dissipation will likely be dependent upon the scale at which the comparison is made. At the local scale of an active hydrothermal manifestation, the fumarolic output will likely overwhelm the soil diffuse flux, while the latter will most likely dominate at a larger scale (at the scale of an entire volcanic complex). For example, based on results from 20 analyzed hydrothermal areas, Harvey et al. (2015) concluded that the contribution of focused venting to the total CO_2_ emission is typically less than 10 %. The TDL, with the ability to characterize the fumarolic CO_2_ output, promises to contribute a substantial advancement in this field in the following years.

## Conclusions

We estimated the total CO_2_ emissions from the main thermal manifestations of Furnas volcano by jointly using two different techniques: the GasFinder 2.0 tunable diode laser and the accumulation chamber method. We find that, in the most vigorously degassing areas, the soil CO_2_ flux contribution (approximately 9.2 t day^−1^) represents a minor (~18 %) contribution to the total CO_2_ output, which is dominated by the fumaroles (about ~50 t day^−1^). The CO_2_ output contributed by the fumaroles is larger than that contributed by Furnas springs (~12 t day^−1^, Cruz et al. 1999), but far lower than the total hydrothermal diffuse degassing flux (~968 t day^−1^) at the scale of the entire volcano. This observation supports the conclusions that although fumaroles are the most visible surface manifestations of thermal activity, they are not necessarily the biggest contributors to the total CO_2_ output from quiescent, Solfatara-stage volcanoes, where CO_2_ is mainly released in silent, invisible form through soil emissions.

Summing up the CO_2_ flux contributions for the fumarolic emissions, the degassing soils and the springs (12 t day^−1^, Cruz et al. 1999), the total volcanic/hydrothermal CO_2_ output for Furnas volcano is estimated to be ~1030 t day^−1^. These results show once more the importance of taking into consideration both soil degassing and gas vent emissions to estimate the CO_2_ emission in hydrothermal areas, since their relative contribution seems to be quite different depending on the study sites. Using a portable Multi-GAS, we also obtained the CO_2_/H_2_S ratio signature for the investigated fumaroles, and concluded that the Furnas fumaroles are, in their present state of activity, weak, but yet not negligible, sources of H_2_S (~0.25 t day^−1^).

## Additional file

Additional file 1:
**The supplementary file is a more detailed documentation about the TDL acquisitions and data elaboration.** A1. CO_2_ TDL-datasets. A2. Parameters used to perform sGs with CO_2_ concentrations TDL data and Zonal Statistic on E-Type maps. A3. Parameters used to perform sGs with soil CO_2_ flux data (accumulation chamber). In A1 section, each path laser-retroreflector acquisition during the campaigns carried out at Furnas Lake (Additional file 1: Table S1) and Furnas Village (Additional file 1: Table S2) is shown. In A2 section, more details about statistical approach and elaboration of data to create the distribution CO_2_ concentration maps are shown (Additional file 1: Tables S3, S4, and S5). Finally, parameters used to perform sGs with soil CO_2_ flux data are shown in A3 section (Tables S6 and S7).
