# Supplementary material for: Total (fumarolic + diffuse soil) CO2 output from Furnas volcano
Source: Earth Planets Space. 2015 Oct 26;67(1):174. doi: 10.1186/s40623-015-0345-5 (PMC5012352; doi:10.1186/s40623-015-0345-5)
Supplement: Additional file 1: — The supplementary file is a more detailed documentation about the TDL acquisitions and data elaboration. A1. CO2 TDL-datasets. A2. Parameters used to perform sGs with CO2 concentrations TDL data and Zonal Statistic on E-Type maps. A3. Parameters used to perform sGs with soil CO2 flux data (accumulation chamber). In A1 section, each path laser-retroreflector acquisition during the campaigns carried out at Furnas Lake (Additional file 1: Table S1) and Furnas Village (Additional file 1: Table S2) is shown. In A2 section, more details about statistical approach and elaboration of data to create the distribution CO2 concentration maps are shown (Additional file 1: Tables S3, S4, and S5). Finally, parameters used to perform sGs with soil CO2 flux data are shown in A3 section (Tables S6 and S7). [file 40623_2015_345_MOESM1_ESM.pdf]

Total (fumarolic + diffuse soil) CO_2 output from Furnas Volcano

Pedone M*[1,2], Viveiros F[3], Aiuppa A[1,2], Giudice G[2], Grassa F[2], Gagliano AL[2] Francofonte V[2], Ferreira T[3]

A1. CO_2 TDL-datasets

The dataset acquired at the different investigated area during the field campaigns is reported in the next tables (Tabs. S1 and S2). In the tables, each row refers to a specific laser-mirror open-path configuration. The following parameters are reported in each table:

N: progressive number of the path;

Path: code identifying each GasFinder-retro-reflector path. Letters indicate position of the laser unit and numbers refer to position of the mirrors (e.g.: “A1” is the open path linking the “A” position of Laser and the “1” position of retro-reflector).

Dist: the optical path length (the distance between the GasFinder unit and the retro-reflector), in meters.

M: average CO_2 concentration (ppm).

Std. Dev: standard deviation of CO_2 concentrations.

Table S1. Furnas Lake TDL dataset. "DIST" is the optical path length (the distance between the GasFinder unit and the retro-reflector), in meters. "M" is the average CO_2 concentration, in ppm.

N	PATH	DIST (m)	M (ppm)	STD. DEV
1	A1	39	594	84.65
2	A2	56	692	103.9
3	A3	62	534	51.88
4	B1	44	595	44.20
5	B2	61	615	99.98
6	B3	60.5	574	90.23
7	C3	62	662	36.61
8	C2	66	605	81.50
9	C1	51	532	61.06
10	D1	60	463	56.56
11	D2	71	499	91.91
12	D3	65	493	76.88
13	E3	68	896	123.7
14	E2	78	400	36.92
15	E1	68	635	95.76
16	A6	43	579	63.08
17	A5	48	508	28.31
18	A4	56	615	35.13
19	B4	53	548	78.68
20	B5	41	585	45.22
21	B6	34	485	37.61
22	C6	29	415	20.20
23	C5	37	522	24.48
24	C4	53	790	132.0
25	D4	55	601	101.1
26	D5	33	422	40.23
27	D6	27	455	4.370
28	E4	57	545	72.90
29	E5	31	504	72.21
30	E6	23	500	10.46

Table S2. Furnas Village TDL dataset. "DIST" is the optical path length (the distance between the GasFinder unit and the retro-reflector), in meters. "M" is the average CO_2 concentration, in ppm.

N	PATH	DIST (m)	M (ppm)	STD. DEV
1	A1	51	564	46.50
2	A2	61	596	21.96
3	A3	59	599	63.70
4	B1	51	592	50.34
5	B2	59	662	79.84
6	B3	57	594	39.79
7	C1	46	679	56.76
8	C2	54	617	40.02
9	C3	51	573	72.96
10	D1	37	533	57.82
11	D2	43	601	58.55
12	D3	38	622	79.27
13	E1	36	654	75.74
14	E2	37	589	31.86
15	E3	29	549	60.11
16	A4	34	498	77.58
17	A5	43	527	35.34
18	A6	59	612	45.97
19	B4	34	600	58.41
20	B5	44	653	129.2
21	B6	56	682	39.17
22	C4	31	683	68.49
23	C5	40	702	78.78
24	C6	51	636	62.84
25	D4	27	851	35.45
26	D5	32	708	77.39
27	D6	34	622	28.78
28	E4	36	539	46.81
29	E5	35	538	36.72
30	E6	27	434	26.92

A2. Parameters used to perform sGs with CO_2 concentrations TDL data and Zonal Statistic on E-Type maps

Table S3. Parameters used to perform sGs with Furnas Lake CO_2 concentrations TDL data.

**Data transformation:**	Normal score
**Number of realizations to generate:**	100
**Interpolation method:**	Simple Kriging
**Number of simulated nodes to use:**	12
**Assign data to nodes:**	yes
**Cell size:**	1 m
**Variogram model:**	Spherical
**Variogram criteria:**	Nugget: 0.45; sill: 1; range: 29 m

Table S4. Parameters used to perform sGs with Furnas Village CO_2 concentrations TDL data.

**Data transformation:**	Normal score
**Number of realizations to generate:**	100
**Interpolation method:**	Simple Kriging
**Number of simulated nodes to use:**	12
**Assign data to nodes:**	yes
**Cell size:**	1 m
**Variogram model:**	Gaussian
**Variogram criteria:**	Nugget: 0.54; sill: 0.62; range: 19 m

Table S5. Zonal Statistic of the CO_2 computed on the distribution maps at Lake (Fig. 4) and Village (Fig. 5) area, and performed by using the Spatial Analyst tool, ArcMap 9.3 (ESRI). Minimum, Maximum, Mean and Standard Deviation of CO_2 concentration values are in ppm. Areas are expressed in m^2.

Site	N. cell	Dim. cel	Area	Min	Max	Mean	St. Dev
Lake	2471	1x1	2471	399.5	895.5	552.4	52.1
Village	1421	1x1	1421	434.3	850.5	605.4	47.4

A3. Parameters used to perform sGs with soil CO_2 flux data (accumulation chamber)

Table S6. Parameters used to perform sGs with Furnas Lake soil CO_2 flux data.

**Data transformation:**	Normal score
**Number of realizations to generate:**	100
**Interpolation method:**	Simple Kriging
**Number of simulated nodes to use:**	12
**Assign data to nodes:**	yes
**Cell size:**	3 m
**Variogram model:**	Spherical
**Variogram criteria:**	Nugget: 0.58; sill: 1.03; range: 14 m

Table S7. Parameters used to perform sGs with Furnas Village soil CO_2 flux data.

**Data transformation:**	Normal score
**Number of realizations to generate:**	100
**Interpolation method:**	Simple Kriging
**Number of simulated nodes to use:**	12
**Assign data to nodes:**	yes
**Cell size:**	3 m
**Variogram model:**	Gaussian
**Variogram criteria:**	Nugget: 0.8; sill: 1.1; range: 35 m
